# Quadruple Primary Malignancies over 2 Years with Germline Mutation in Krebs Cycle Enzyme Gene Fumarate Hydratase

**DOI:** 10.1155/2024/5591237

**Published:** 2024-06-06

**Authors:** Solaleh Aminian, Fawaz Al-Alloosh, Fatemeh Yadegari, Shiva Zarinfam, Haider Hamza Al-Abedi, Keivan Majidzadeh-A.

**Affiliations:** ^1^Genetics Department, Breast Cancer Research Center, Motamed Cancer Institute, ACECR, Tehran, Iran; ^2^Warith International Cancer Institute, Karbala, Iraq

## Abstract

Multiple primary cancers (MPCs) are defined as the presence of more than one cancer in an individual that is not due to recurrence, metastasis, or local spread. Different factors such as copathogenic genetic mutations, environmental factors, lifestyle, and first cancer treatment increase the possible occurrence of subsequent malignancies. In recent years, the risk of MPCs has increased due to improved treatment; however, quadruple primary malignancies are still rare and require further investigation and treatment of the underlying cause. Here, we present a 64-year-old man with a 40-year history of cigarette smoking who developed quadruple primary malignancies of the epiglottis, kidney, pancreas, and lung. To investigate the possible genetic cause, we performed WES, and a variant of c.580G > A (Ala194Thr) was discovered in exon 5 of the Krebs cycle enzyme gene, fumarate hydratase (FH). This substitution was classified as VUS in Clinvar and likely pathogenic by Varsome and Franklin software. The structural analysis showed that the variation found was localized in a highly conserved alpha helix in the D2 domain near the FH hinge region (<6 Å), suggesting that enzyme activity was affected by a perturbation in protein quaternary structure. Because of the well-established role of FH mutations in renal cancer risk, it was possible that the FH mutation could have led to the development of renal cell carcinoma in this case. The biological mechanisms of MPCs suggest that subsequent primary malignancies are triggered by the combined effects of environmental factors, such as smoking and genetics.

## 1. Introduction

Multiple primary cancers (MPCs) are defined as two or more histologically distinct malignancies occurring in an individual and not due to spread, recurrence, or metastasis. MPCs were first described as a rare phenomenon by Billroth in 1889 [1]; today, however, the improved survival of cancer patients has increased the risk of developing MPCs. Depending on the time interval between the diagnosis of the first and second primary malignancy, MPCs are classified as metachronous (more than 6 months apart) or synchronous (at the same time or within 6 months of each other) [2]. Although the mechanism of MPCs is not fully understood, several factors, including certain types of treatment such as radiotherapy and chemotherapy, lifestyle behaviors such as smoking and dietary habits, and genetic factors, can influence the development of subsequent malignancies [3–7]. In this study, we present an extremely rare case of a 64-year-old man with a germline mutation in the gene encoding fumarate hydratase (FH), leading to four primary malignant tumors. FH is a homotetrameric enzyme of the tricarboxylic acid cycle (TCA cycle) that catalyzes reversible hydration/dehydration of fumarate to malate. FH is an unusual example of a gene in which heterozygous or homozygous germline mutations cause distinct pathologic phenotypes. Heterozygous mutations in the FH are responsible for susceptibility to hereditary leiomyomatosis and renal cell carcinoma (HLRCC). HLRCC is an autosomal dominant in which affected persons show cutaneous manifestations, uterine fibroids, uterine leiomyosarcoma, and highly aggressive kidney cancers. Leiomyomas of the skin and uterus are the most common features of HRCC with nearly complete penetrance. Uterine fibroid development accounts for approximately all affected young women, 70 to 80% of whom will undergo either a myomectomy or hysterectomy. Homozygous or compound heterozygous germline FH mutations lead to fumarase deficiency (FHD) causing neurological impairment, infant-onset seizures, muscular hypotonia, facial dysmorphism, developmental delay, and atrophic brain. Interestingly, in 2021, Kamihara and colleagues showed that individuals with FH PV (FH pathogenic variant) developed kidney cancer more than those with FH-d PV (FH deficient pathogenic variant) (odds ratio, 9.0; 95% CI, 4.4 to 20.0; *P* < 0.001). In HLRCC, individuals typically develop light brown papules on their trunk, extremities, and occasionally on the face around age 25. Uterine leiomyoma manifests as irregular or heavy menstrual bleeding or pelvic pain at 18 to 53 years old. It is crucial to note that RCCs correlated with FH are mostly unilateral non-clear-cell, very aggressive, and tend to metastasize sooner. The mean age of RCC diagnosis is 40 [8]. More recent studies have opened the possibility of monoallelic FH mutation giving rise to pheochromocytomas or paraganglioma.

## 2. Method

**Figure 1:**
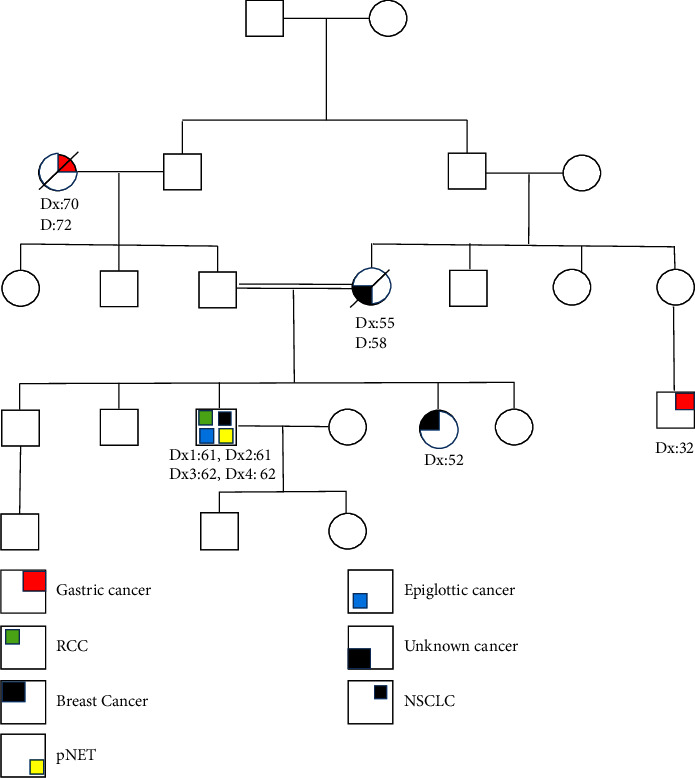
The pedigree information of this family.

The process of genetic counseling was conducted for a male patient aged 64 years, who had been diagnosed with four primary cancers, namely, differentiated squamous cell carcinoma of the epiglottis, renal cell carcinoma (RCC), pancreatic neuroendocrine tumor (pNET), and non-small-cell lung cancer (NSCLC). After signing an informed consent form from the proband, pedigree information, including consanguinity, affected family members, associated diseases, and family history, was collected through interviews (Figure 1). To investigate the genetic cause, we performed whole exome sequencing (WES) on extracted DNA from peripheral blood leukocytes. Subsequently, structural analysis was performed to predict the structural consequences of the amino acid substitution detected identified through WES.

## 3. Result

In this study, we present a 64-year-old Iraqi man with a history of four primary malignant tumors who had been admitted to the Cancer Institute for genetic counseling in December 2022. He had a history of hypertension and type 2 diabetes. Also, he has a 40-year history of cigarette smoking and has recently reduced to 1 pack/day. His family history was notable for an unknown cancer in his mother aged 55, his brother in his 10s with a sarcoma, and a sister recently diagnosed with breast cancer (Figure 1). In summary, in December 2019, he was admitted to the hospital with a cough, chest pain, and difficulty breathing. Computer tomography (CT) showed a defined mass in the supra-glottic larynx, subsequently confirmed as a moderately differentiated squamous cell carcinoma of the epiglottis. Simultaneously, PET-CT also showed increased metabolic uptake in the lower calyx of the left kidney, which was subsequently confirmed as primary papillary renal cell carcinoma. He underwent a partial nephrectomy followed by radio-chemotherapy (Gemcitabine, Cisplatin). A year later (2022), the patient presented with two primary cancers, including pancreatic neuroendocrine tumor (pNET) and non-small-cell lung cancer (NSCLC), which were diagnosed almost at the same time. At first, in January 2022, computed tomography (CT) of the abdomen showed a mass shadow in the head of the pancreas with a diameter of 30 mm. The histopathological examination results confirmed that the pancreatic mass was a neuroendocrine tumor. Later, in March 2022, after a chest CT scan and a transbronchial lung biopsy, the diagnosis of primary NSCLC was made and the patient started chemotherapy.

### 3.1. Genetics Analysis

After undergoing genetic counseling and collection of personal and familial medical data, DNA extraction was carried out, followed by targeted sequencing using Next-Generation Sequencing (NGS) technology. We found a candidate genetic variation [c.580 G > A, p. Ala194Thr] in the gene encoding the Krebs cycle enzyme fumarate hydratase (FH; NM_000143.4). This substitution was classified as VUS in Clinvar and likely pathogenic in Varsome and Franklin.

### 3.2. Molecular Modelling of Ala194Thr

FH is a homotetramer protein with each monomer consisting of three domains termed D1 (residues: 49–188), D2 (residues: 189–439), and D3 (residues: 440–510). The D2 domain is at the core of the tetrameric enzyme and facilitates tetramerization. D1 and D3 domains are arranged at the corners of the homotetramer to form the entrance of the four fumarase active sites [9]. Shorthouse et al. have investigated all possible variants of FH. They subsequently classified the FH potentially disruptive mutations into three groups: (I) binding site-associated mutations, (II) hinge-associated mutations, and (III) destabilizing mutations [10]. They considered mutations with alpha-carbon (CA) atoms within 6 Å of any residue in the binding site or hinge region as potentially harmful due to disruption of the protein dynamics and function. The FH gene VUS detected in our study is assumed to affect enzyme activity for two reasons. Firstly, evolutionary conservation analysis of amino acid residues showed that Ala194 is conserved among FH proteins across various species. Secondly, according to Shorthouse et al.'s predictions, the modified Ala194 residues were situated close to the hinge region of FH, within a distance of less than 6 Å. This suggests that the enzyme lost functionality because of a perturbation in the protein's dynamics.

## 4. Discussion

We reported a 64-year-old male patient who developed four primary malignancies, including squamous cell carcinoma of the epiglottis, RCC, pNET, and NSCLC, in two years. He is a current cigarette smoker with a history of hypertension and type 2 diabetes. To investigate the possible genetic cause, we performed targeted sequencing, which revealed the presence of a heterozygous variant c.580G > A (Ala194Thr) within exon 5 of the FH gene. This gene encodes an enzyme involved in the Krebs cycle. Heterozygous FH germline mutations lead to HLRCC. Individuals affected by this condition possess a genetic predisposition to develop uterine fibroids, uterine leiomyosarcoma, and highly aggressive renal cancers [11, 12]. The variant found in our case, Ala194Thr, was classified as VUS in Clinvar but as likely pathogenic in Franklin and Varsome (using ACMG criteria). Structural analysis showed that the altered Ala194 residues were located close to the hinge region of FH, suggesting that enzyme activity was affected by a perturbation in protein quaternary structure [10]. This particular case underwent radiation therapy and exhibited smoking behavior. Although a notable association between the risk of kidney cancer and FH germline mutations is evident [11, 12], it cannot be disregarded that lifestyle choices, especially smoking, may also contribute to the onset of kidney cancer [13]. Pancreatic neuroendocrine tumors (pNETs) are the third cancer diagnosed in our patient. There is no confirmed association between pNETs and environmental factors such as cigarette smoke [13]. We were inquiring as to whether the FH mutation can have an impact on the development of pNETs in this specific patient. Through our investigation of relevant literature, we have discovered a recent identification of a positive correlation between the mutation in the FH gene and the occurrence of pheochromocytoma or paraganglioma (PHEO/PGL) [14, 15]. In most cases, peripancreatic PGLs were misdiagnosed preoperatively as pNETs [16–18]. Assuming the accuracy of the diagnosis of pNETs in this patient, it is uncertain whether the observation of pNETs in this patient was incidental or if pNETs are also part of the FH-associated tumor spectrum. It is worth mentioning that a recent study has revealed a correlation between mutations in the Krebs cycle enzyme succinate dehydrogenase (SDHD) and the occurrence of pNETs [19]. We hereby propose future studies to investigate the association between mutations in another Krebs cycle enzyme, fumarate hydratase (FH), and pNETs.

In light of the aggressiveness and poor prognosis associated with FH-related RCC, surgical excision of even small RCCs and their adjacent lymph nodes should be considered. Uterine leiomyomas could be managed surgically (myomectomy and hysterectomy) or medically (gonadotropin-releasing hormone agonists (GnRHa) and intrauterine devices releasing progesterone). Cutaneous leiomyomas may be eliminated through carbon dioxide laser, cryotherapy, or surgical excision.

## Data Availability

The reference sequence (NM_000143.4) and 3D structure (3E04.pdb) of FH were obtained from the National Center for Biotechnology Information, NCBI (https://www.ncbi.nlm.nih.gov/), and Protein Data Bank, PDB (https://www.rcsb.org/), respectively. The classification of variants was determined according to the Clinvar database at NCBI (https://www.ncbi.nlm.nih.gov/clinvar/).

## Abbreviations

MPCs: Multiple primary cancers
FH: Fumarate hydratase
RCC: Renal cell carcinoma
pNET: Pancreatic neuroendocrine tumor
NSCLC: Non-small-cell lung cancer.
